# Low expression of ANT1 confers oncogenic properties to rhabdomyosarcoma tumor cells by modulating metabolism and death pathways

**DOI:** 10.1038/s41420-020-00302-1

**Published:** 2020-07-24

**Authors:** J. Vial, P. Huchedé, S. Fagault, F. Basset, M. Rossi, J. Geoffray, H. Soldati, J. Bisaccia, M. H. Elsensohn, M. Creveaux, D. Neves, J. Y. Blay, F. Fauvelle, F. Bouquet, N. Streichenberger, N. Corradini, C. Bergeron, D. Maucort-Boulch, P. Castets, M. Carré, K. Weber, M. Castets

**Affiliations:** 1 Cell death and Childhood Cancers Laboratory—Equipe labellisée LabEx DEV2CAN, Centre de Recherche en Cancérologie de Lyon, INSERM U1052-CNRS UMR5286, Université de Lyon, Centre Léon Bérard, 69008 Lyon, France; 2 Aix-Marseille Université, Inserm UMR_S 911, Centre de Recherche en Oncologie biologique et Oncopharmacologie, Faculté de pharmacie, Marseille, France; 3 Department of Cell Physiology and Metabolism, University of Geneva, CMU, CH-1211 Geneva, Switzerland; 4 Service de Biostatistique—Bioinformatique, Pôle Santé Publique, Hospices Civils de Lyon, F-69003 Lyon, France; 5 Netris Pharma, Lyon, France; 6 Université Grenoble Alpes, INSERM, US17, MRI facility IRMaGe, 38000 Grenoble, France; 7 Roche Institute, Boulogne-Billancourt, France; 8 Hospices Civils de Lyon, Lyon, France; 9 INMG CNRS UMR 5310, INSERM U1217, Université Claude Bernard Lyon, Lyon, France

**Keywords:** Paediatric cancer, Cell death

## Abstract

Rhabdomyosarcoma (RMS) is the most frequent form of pediatric soft-tissue sarcoma. It is divided into two main subtypes: ERMS (embryonal) and ARMS (alveolar). Current treatments are based on chemotherapy, surgery, and radiotherapy. The 5-year survival rate has plateaued at 70% since 2000, despite several clinical trials. RMS cells are thought to derive from the muscle lineage. During development, myogenesis includes the expansion of muscle precursors, the elimination of those in excess by cell death and the differentiation of the remaining ones into myofibers. The notion that these processes may be hijacked by tumor cells to sustain their oncogenic transformation has emerged, with RMS being considered as the dark side of myogenesis. Thus, dissecting myogenic developmental programs could improve our understanding of RMS molecular etiology. We focused herein on ANT1, which is involved in myogenesis and is responsible for genetic disorders associated with muscle degeneration. ANT1 is a mitochondrial protein, which has a dual functionality, as it is involved both in metabolism via the regulation of ATP/ADP release from mitochondria and in regulated cell death as part of the mitochondrial permeability transition pore. Bioinformatics analyses of transcriptomic datasets revealed that ANT1 is expressed at low levels in RMS. Using the CRISPR-Cas9 technology, we showed that reduced ANT1 expression confers selective advantages to RMS cells in terms of proliferation and resistance to stress-induced death. These effects arise notably from an abnormal metabolic switch induced by ANT1 downregulation. Restoration of ANT1 expression using a Tet-On system is sufficient to prime tumor cells to death and to increase their sensitivity to chemotherapy. Based on our results, modulation of ANT1 expression and/or activity appears as an appealing therapeutic approach in RMS management.

## Introduction

Childhood cancers may be considered as aberrations of pre- and postnatal ontogeny, resulting from dysregulated developmental processes^1,2^. Development is characterized by rapid proliferative expansion of cells, their migration along appropriate routes towards organs, tissue refinement by elimination of excessive cells, and the terminal differentiation of survivors into normal cell types. In particular, during development, cells in excess are secondarily pruned through switches in developmental programs governing survival; the regulation of cell suicide pathways is then crucial. It has notably been postulated that during early embryogenesis, a pool of myoblasts is generated in somites during skeletal muscle development^3^. A fraction of these cells undergoes differentiation, another subset exits cell cycle to constitute a niche of resting stem cells, while the remaining are likely eliminated through different regulated forms of cell death^3^. During development, some supernumerary cells resist these death signals as a potential first pathological step towards cancer^1^. This tumor resistance to cell death also plays a key role in constitutive or acquired resistance to treatments^4^.

Rhabdomyosarcoma (RMS) is the most frequent form of pediatric soft-tissue sarcoma, accounting for 5% of solid pediatric tumors, with a 5-year survival rate that caps at 60–70%^5,6^. Molecular bases of RMS remain unclear, notably in the sizeable fraction of translocation negative tumors. Regarding cell death resistance, the prognosis has been correlated with the expression of Bcl family members in patients and resistance to apoptosis linked to failure of conventional therapies in RMS cell lines. However, robust characterization of cell death pathways altered in RMS is still lacking^5,7^. RMS are thought to arise from malignant transformation of muscle precursors^6^. Embryonal RMS (ERMS), so-called because they resemble embryonic muscle, present various degrees of myogenic differentiation, ranging from small round cells to larger oblong ones, sometimes having a strap-like appearance for the most differentiated ones, occasionally with cross striations and multinucleation^6^. Aside from these morphological observations supporting the myoblast-like nature of tumor cells, RMS expression signatures are also characterized by widespread expression of embryonic muscle-specific markers such as myogenin, desmin, or MyoD. Moreover, the notion that the molecular pathogenesis of RMS shares similarities with processes involved in myogenesis or mechanisms that have gone awry in muscular dystrophies has begun to emerge^8–10^. Besides gold standard effectors of death pathways, we hence hypothesized that genes involved in specific cell survival/death imbalance during myogenesis or in the etiology of muscular dystrophies may also play a role in RMS tumorigenesis.

Based on exhaustive bioinformatics-driven bibliographic data mining, we identified adenine nucleotide translocator 1 (ANT1) as a potent player in RMS. ANT1 is the heart- and muscle-specific isoform of the ANT mitochondrial inner membrane protein family. ANT1 plays a role in myoblast differentiation^11^ and is associated with mitochondrial myopathy and cardiomyopathy^12–14^. ANT1 regulates ATP/ADP exchange across the mitochondrial inner membrane. Indeed, it exports ATP produced by OXPHOS metabolism to the cytoplasm to power cellular reactions and simultaneously imports ADP to restore intra-mitochondrial stock. ANT1 was also initially described as a key component of the so-called mitochondrial permeability transition pore (mPTP), a solute channel assembled at the junction between the inner and the outer mitochondrial membrane whose opening triggers mitochondrial permeability transition (MPT)^15–17^. Although this view was challenged^18^, pharmacological and genetic inactivation of ANT family members have strengthened the idea that ANT proteins are indeed involved, at least to some extent, in mPTP formation/regulation^19–22^. mPTP opening drives osmotic influx of water into mitochondrial matrix, resulting in the collapse of these organelles. This ultimately leads to execution of regulated cell death, whether it be MPT-driven necrosis or apoptosis, the latest being induced by the release of the cytochrome c from the mitochondrial intermembrane space and the subsequent activation of executioner caspases^19,21–23^. ANT1 is hence considered to be at the crossroad of several cell death and metabolic signaling pathways. Here, we show that ANT1 encoding gene, *SLC25A4* (further referred to as *ANT1*), is expressed at low levels in RMS tumors. Using the inducible CRISPR-Cas9 strategy, we establish that downregulation of *ANT1* expression in RMS cells increases both their proliferation and their resistance to stress-induced cell death. Importantly, restoring *ANT1* expression is sufficient to counteract resistance to cell death and to increase sensitivity of tumor cells to chemotherapy. Hence, we unveil a potent role for ANT1 as a new tumor suppressor in RMS.

## Results

### Low levels of *ANT1* expression in RMS favor tumor cell proliferation

**Fig. 1:**
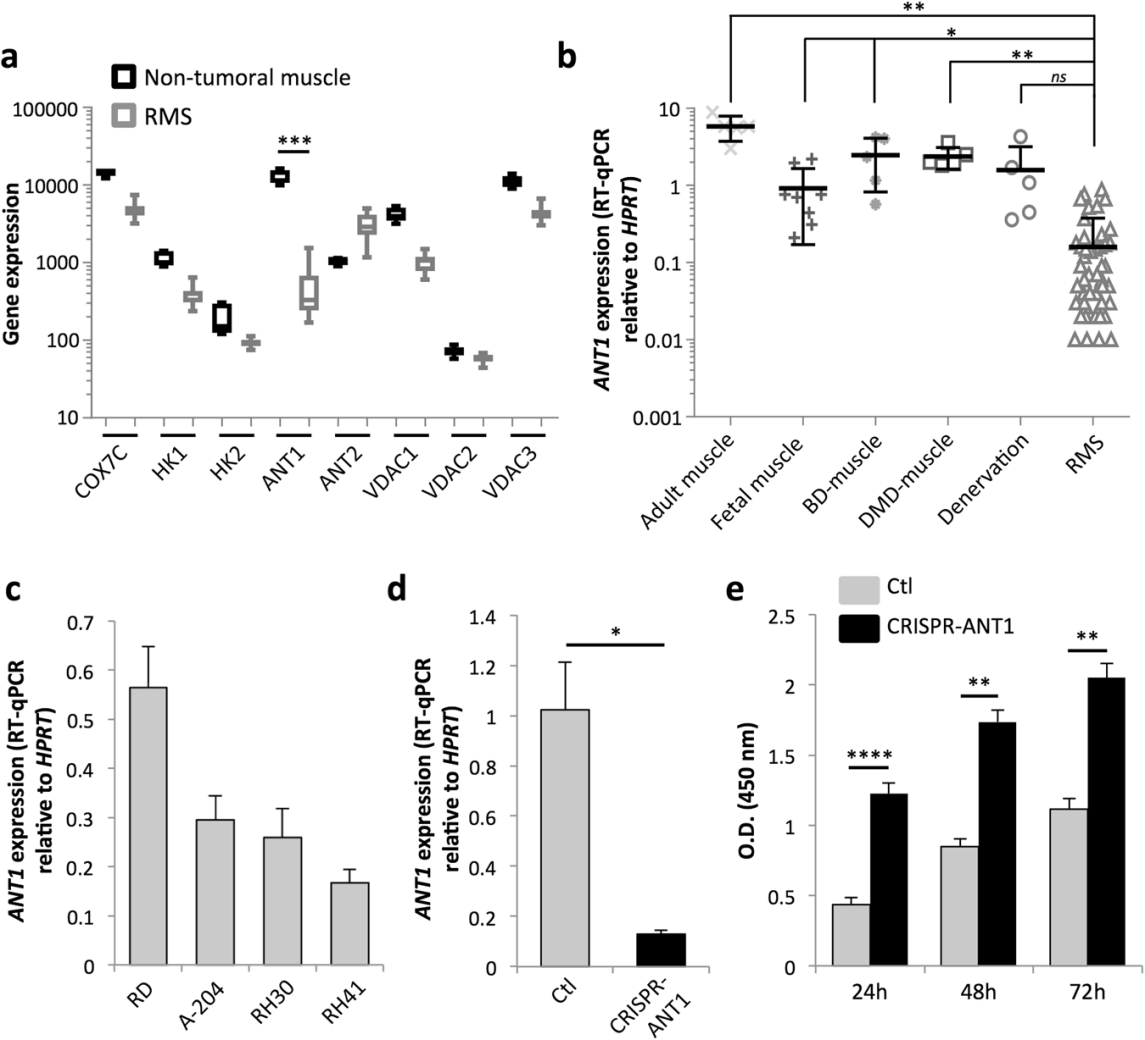
Low *ANT1* expression in RMS favors tumor cell proliferation. **a** Bioinformatics analysis of the expression of the *ANT1* gene (*SLC25A4*) and of some of its partners in a cohort of non-tumoral muscle (*n* = 6), ERMS (*n* = 8), and ARMS (*n* = 10) from the GSE28511 transcriptomic dataset. ***p < 0.001; two-sided independent samples *T*-test. **b** Quantification of *ANT1* gene (*SLC25A4*) expression by RT-qPCR, relative to the housekeeping gene *HPRT* in RMS biopsies (*n* = 67), normal adult muscle (*n* = 5), fetal muscle (*n* = 9), and in biopsies from patients with muscle weakness (BD Becker dystrophy, DMD Duchenne muscular dystrophy, *n* = 5 each). *p < 0.05, **p < 0.01, ns not significant; two-sided independent samples *T*-test. **c** Quantification of *ANT1* expression by RT-qPCR, relative to the housekeeping gene *HPRT* in ERMS cell lines (RD and A-204), and ARMS cell lines (RH30 and RH41). Results are presented as means ± s.d.; *n* = 3. **d** Efficiency of *ANT1* silencing by CRISPR-Cas9 in RD cells, 48 h after doxycycline treatment. Quantification of *ANT1* expression by RT-qPCR, relative to the housekeeping gene *HPRT*. Results are presented as means ± s.d.; *n* = 3. *p < 0.05; two-sided independent samples *T*-test. **e** Increase in number of metabolically active cells as measured by WST-1 assay in RD^Low^ cells, at different time points after silencing of *ANT1* expression by doxycycline treatment. Results are presented as means ± s.d.; *n* = 3. **p < 0.01, ****p < 0.0001; two-sided independent samples *T*-test.

Changes in *ANT1* expression have been reported in several disorders and are notably associated with muscular defects^24–27^. However, its expression profile in cancers has so far barely been studied, especially in RMS. To analyze the expression levels of *ANT1* in RMS, we first performed a bioinformatics analysis of the publicly available GSE28511 dataset, dedicated to the comparison of normal skeletal muscle tissue (*n* = 6) versus RMS (*n* = 8 ERMS and *n* = 10 ARMS). We observed that the expression of *ANT1* is significantly lower (24-fold reduction) in RMS than in non-tumoral skeletal muscle (threefold reduction at the most for the electron transport chain protein COX7C or the mitochondrial hexokinase HK1) (Fig. 1a). As previously reported in other cancers, the expression of the ANT2 encoding gene, *SLC25A5*, increased threefold in tumors^28,29^. We confirmed this result by RT-qPCR, by showing that the level of *ANT1* expression was higher in adult, fetal, and dystrophic muscles than in 67 pediatric RMS samples (Fig. 1b). Using the E-TABM-1202 dataset, we observed that high *ANT1* expression levels tend to be positively associated with a better outcome in fusion-negative RMS, although this observation needs to be confirmed on a larger cohort (Fig. S1a).

We hypothesized that low *ANT1* expression may confer a selective advantage to tumor cells. Using the R2 cancer software, we observed that *ANT1* expression in patients tends to be negatively correlated with the expression of *CDC7*, which has been described as an inducer of smooth muscle cell proliferation^30^, and of *MKI67*, suggesting that ANT1 may influence cell proliferation (Fig. S1b, c). To investigate whether ANT1 impacts cell proliferation, we screened its expression in different RMS cell lines by RT-qPCR (Fig. 1c). Based on this screen, we selected the ERMS RD cell line, which displayed the highest *ANT1* expression level, to assess the consequences of *ANT1* knock-down in RMS cells. We set up a stable doxycycline-inducible CRISPR-Cas9 system that reduced *ANT1* expression by 88% (Fig. 1d), without affecting *ANT2* expression (Fig. S1d). These cells will be further referred to as RD^Low^. Decreased *ANT1* expression in these cells triggered a 2.8-, 2.1-, and 1.85-fold increase in number of viable cells, 24, 48, and 72-h post-silencing, respectively, as measured by WST-1 assay (Fig. 1e), and a 42.5% increase in viable cell concentration in normal growth conditions (Fig. S1e), without any impact on death induction (not shown). Similar results were observed by silencing ANT1 via siRNA in immortalized myoblasts (Fig. S1f, g). Thus, RMS are associated with low *ANT1* expression, which may sustain tumor cell proliferation.

### Loss of ANT1 confers selective advantage to tumor cells by maintaining them in a proliferative state

**Fig. 2:**
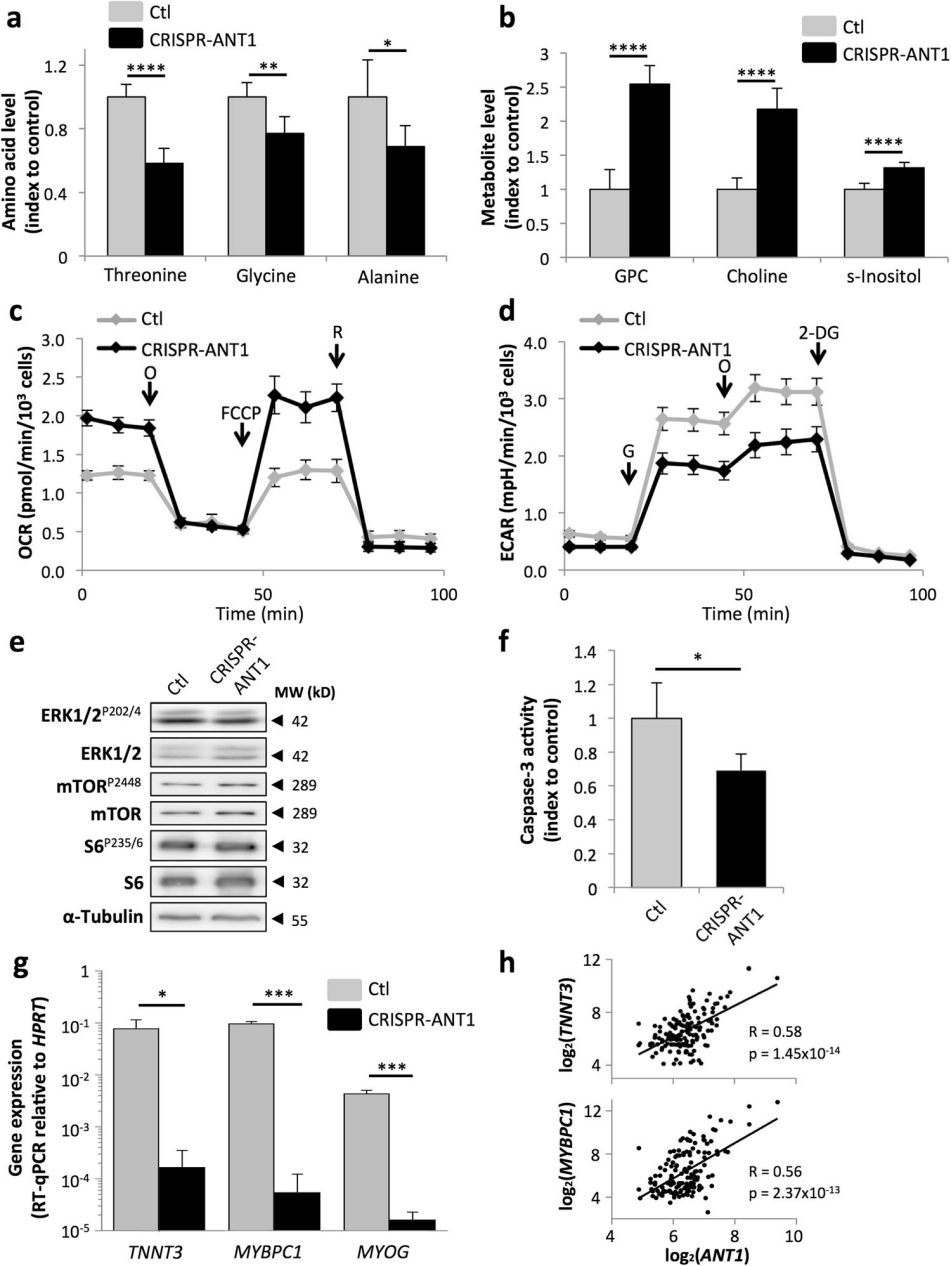
Loss of ANT1 confers selective advantage to tumor cells by maintaining them in a proliferative state. **a** Changes in amino acid abundance in RD^Low^ cells as compared to control cells. Results are presented as means ± s.d. of the ratio between metabolites abundance in RD^Low^ cells compared to control cells; *n* = 5. *p < 0.05, **p < 0.01, ****p < 0.0001; two-sided independent samples *T*-test. **b** Changes in choline derivatives and inositol metabolite abundance in RD^Low^ cells compared to control cells. GPC glycerophosphocholine, s-Inositol *scyllo*-Inositol. Results are presented as means ± s.d. of the ratio between metabolite abundance in RD^Low^ cells compared to control cells; *n* = 5. ****p < 0.0001; two-sided independent samples *T*-test. **c** Oxygen consumption rate (OCR) measured by Seahorse in RD^Low^ cells compared to control cells. O Oligomycin, FCCP Carbonyl cyanide-4-(trifluoromethoxy)-phenylhydrazone, R Rotenone. Results are presented as means ± s.d.; *n* = 3. **d** Extracellular acidification rate (ECAR) in RD^Low^ cells compared to control cells, as a hallmark of glycolysis. G Glucose, O Oligomycin, 2-DG 2-deoxy-glucose. Results are presented as means ± s.d.; *n* = 3. **e** Loss of ANT1 appears to have no impact on activation of ERK1/2, S6, and mTORC1 activation, as shown by their phosphorylation levels on western Blots. Tubulin is used as a loading control. **f** Decrease in basal level of caspase-3 activity in RD^Low^ cells. Results are presented as means ± s.d.; *n* = 3. *p < 0.05; two-sided independent samples *T*-test. **g** Decrease in the expression of differentiation markers *TNNT3*, *MYBPC1*, and *MYOG* in RD^Low^ cells as compared to control cells. Results are presented as means ± s.d. of three independent RT-qPCR. *p < 0.05, ***p < 0.001; two-sided independent samples *T*-test. **h***ANT1* expression in RMS biopsies (*n* = 147) is negatively correlated to two markers of skeletal muscle *TNNT3* and *MYBPC1*. R2 cancer analysis, Davicioni E-TABM-1202 dataset.

As ANT1 is at the crossroad of metabolic, death, and mitogen-activated signals^31^, increase in cell proliferation observed in RD^Low^ cells may have multiple origins. Since ANT1 regulates metabolism by controlling ATP/ADP exchange, we first hypothesized that ANT1 downregulation may trigger metabolic changes sustaining their proliferative capacity. Amino acids especially those linked to the tricarboxylic acid cycle are an alternative source of energy used during cancer cell proliferation^32^. Interestingly, metabolomic analyses revealed significant decrease in amino-acid content in RD^Low^ cells, including glycine (Fig. 2a). A similar decrease in glycine content has previously been associated with increased proliferation in cancer cells^33^. At the same time, we detected that the levels of *scyllo*-Inositol, choline, and glycerophosphocholine were significantly increased in RD^Low^ cells (Fig. 2b). This can be linked to previous results showing that myogenesis is associated to a transient increase in phospholipids metabolism during the myoblasts proliferation phase, followed by a decrease in content during the late myotubes’ differentiation steps^34^. Of note, accumulation of these metabolites is also reminiscent of high choline metabolism, which is a hallmark of cancer cells and was shown to sustain their proliferation by providing them with the necessary membrane precursors^35^. To extend these findings, we examined whether ANT1 downregulation also affected the oxidative capacity of the cells, by impacting the export of ATP coupled to the electron transport chain driving OXPHOS metabolism. Using the Seahorse system, we showed that reduced ANT1 expression increases basal oxygen consumption rate (OCR, Fig. 2c), considered to be a marker of OXPHOS metabolism. It is worth noting that a similar switch to OXPHOS metabolism has been described upon activation and proliferation of myogenic precursors during regeneration^36^. RD^Low^ cells were slightly less glycolytic than controls, with a decrease in basal extracellular acidification rate (ECAR) of 31% (Fig. 2d). The spare respiratory capacity (SRC) measures the extra mitochondrial capacity available in a cell to produce energy under conditions of increased demand and is calculated by the difference between the maximal and basal OCR (Fig. 2c and “Material and methods”). SRC increased slightly when ANT1 levels lowered, indicating that those cells have a greater ability to adapt to metabolic stress. Altogether, these results indicate that lowering *ANT1* expression triggers a metabolic switch in RMS cells, similar to the metabolic state of proliferating muscle precursors, which may sustain the proliferative properties of tumor cells.

To determine whether the effect of *ANT1* downregulation on RMS cell proliferation involves other mechanisms, besides this metabolic switch, we next assessed the status of known pathways related to ANT1 and/or involved in myoblast proliferation. While ANT1 has been reported to modulate ERK1/2 activity, we detected no change in total and phosphorylated forms of ERK1/2 (Tyr 202/204) in RD^Low^ cells compared to control cells. Similar levels of total and phosphorylated (active) mTOR (Ser 2448) and S6 (Ser 235/236) indicated that mTORC1 signaling, a well-known pathway associated with cell growth, was also unaffected in RD^Low^ cells (Fig. 2e).

During myogenesis, activation of caspases, well-known cysteine protease executioners of apoptosis, is required at a “sub-apoptotic” level to engage myoblasts in differentiation^37–39^. Since ANT1 is involved in MPT that can result in apoptosis^40,41^, we wondered whether modulation of its expression may affect proliferation via a caspase-associated signaling cascade. We observed that basal caspase-3 activity was significantly decreased in RD^Low^ cells (Fig. 2f). Consistently, expression of the differentiation marker *MYOG* and the specific skeletal muscle markers *TNNT3* and *MYBPC1* are significantly reduced in these cells compared to control cells (Fig. 2g). Moreover, *TNNT3* and *MYBPC1* tend to be correlated with *ANT1* in RMS patients (R2 cancer analysis, Davicioni E-TABM-1202 dataset, Fig. 2h). This suggests an association between *ANT1* expression and tumor cell differentiation status, which might be mediated by caspase-3 activity.

Altogether, these results indicate that downregulation of *ANT1* expression is sufficient to maintain RMS tumor cells in an immature proliferative state most likely by affecting different cellular pathways and processes.

### Low levels of ANT1 favor resistance of tumor cells to death induced by stress and chemotherapy

**Fig. 3:**
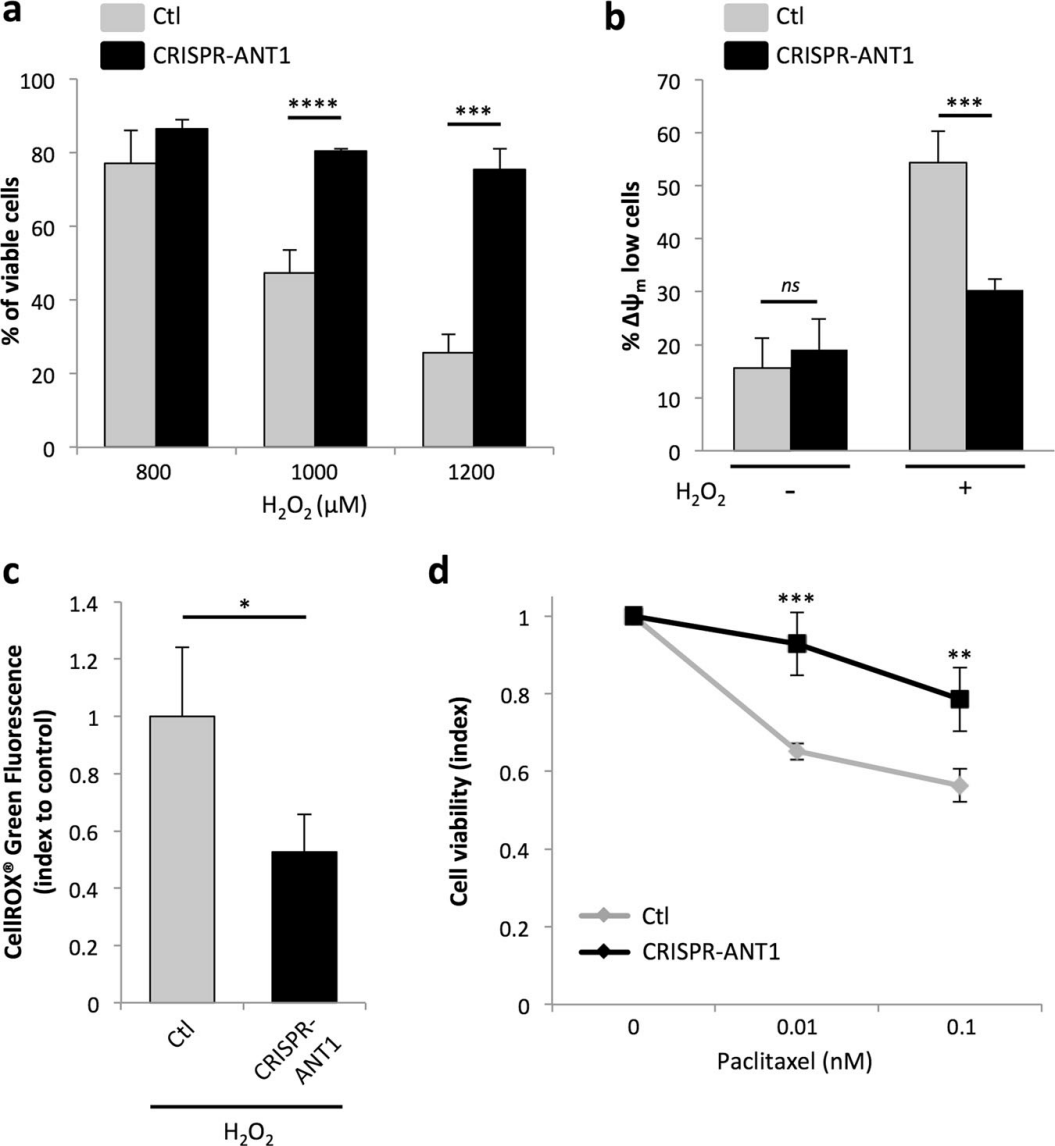
Low levels of ANT1 favor resistance of tumor cells to death induced by stress and chemotherapies. **a** Increase in the percentage of viable cells in RD^Low^ cells treated upon H_2_O_2_ treatment (image cytometry quantification, DAPI/Acridine orange co-staining). Results are presented as means ± s.d.; *n* = 3. ***p < 0.001, ****p < 0.0001; two-sided independent samples *T*-test. **b** Decrease in the cell percentage with loss of mitochondrial membrane depolarization (∆ψ_m_) in RD^Low^ cells upon H_2_O_2_ stress, compared to control cells. Results are presented as means ± s.d.; *n* = 3. ***p < 0.001, ns not significant; two-sided independent samples *T*-test. **c** ROS production is lower in RD^Low^ cells upon H_2_O_2_ stress. ROS production was evaluated by measuring CellROX^®^ fluorescence. Results are presented as means ± s.d.; *n* = 3. *p < 0.05; two-sided independent samples *T*-test. **d** Decreased sensitivity of RD^Low^ cells to Paclitaxel after 24 h of treatment, compared to control cells (WST-1 assay). Results are presented as means ± s.d.; *n* = 3. **p < 0.01, ***p < 0.001; two-sided independent samples *T*-test.

ANT1 exerts a pro-death function notably in conditions of oxidative stress response^42^, as part of the mPTP. We thus decided to study the consequences of its modulation in response to H_2_O_2_ treatment using our CRISPR-Cas9 system. Silencing *ANT1* led to an increase in the percentage of viable RD^Low^ cells compared to control counterparts (Fig. 3a). This effect was accompanied by a loss of mitochondrial membrane potential (∆ψ_m_) (Fig. 3b). H_2_O_2_ triggers death via the generation of reactive oxygen species (ROS). Interestingly, increase in cell viability in RD^Low^ cells was also concomitant to a decrease in ROS production, as measured by monitoring CellROX^®^ fluorescence. This suggests that detoxification of ROS was more efficient in these *ANT1*-silenced cells (Fig. 3c).

Next, we assessed the impact of *ANT1* downregulation on cell sensitivity to chemotherapy. We exposed RD^Low^ cells and controls to increasing concentrations of chemotherapies that are used in the therapeutic management of RMS, such as Paclitaxel and Vincristine, and evaluated their impact on cell viability. As shown on Fig. 3d, *ANT1* silencing reduced the sensitivity of RMS cells to Paclitaxel. Similar results were obtained with Vincristine (Fig. S1h). Hence, these results indicate that, aside from promoting cell proliferation, reduction in *ANT1* expression is also sufficient to increase the resistance of RMS tumor cells to death, thereby conferring a second selective advantage to these cells.

### Restoration of ANT1 is sufficient to sensitize RMS cells to death induced by stress and chemotherapy

**Fig. 4:**
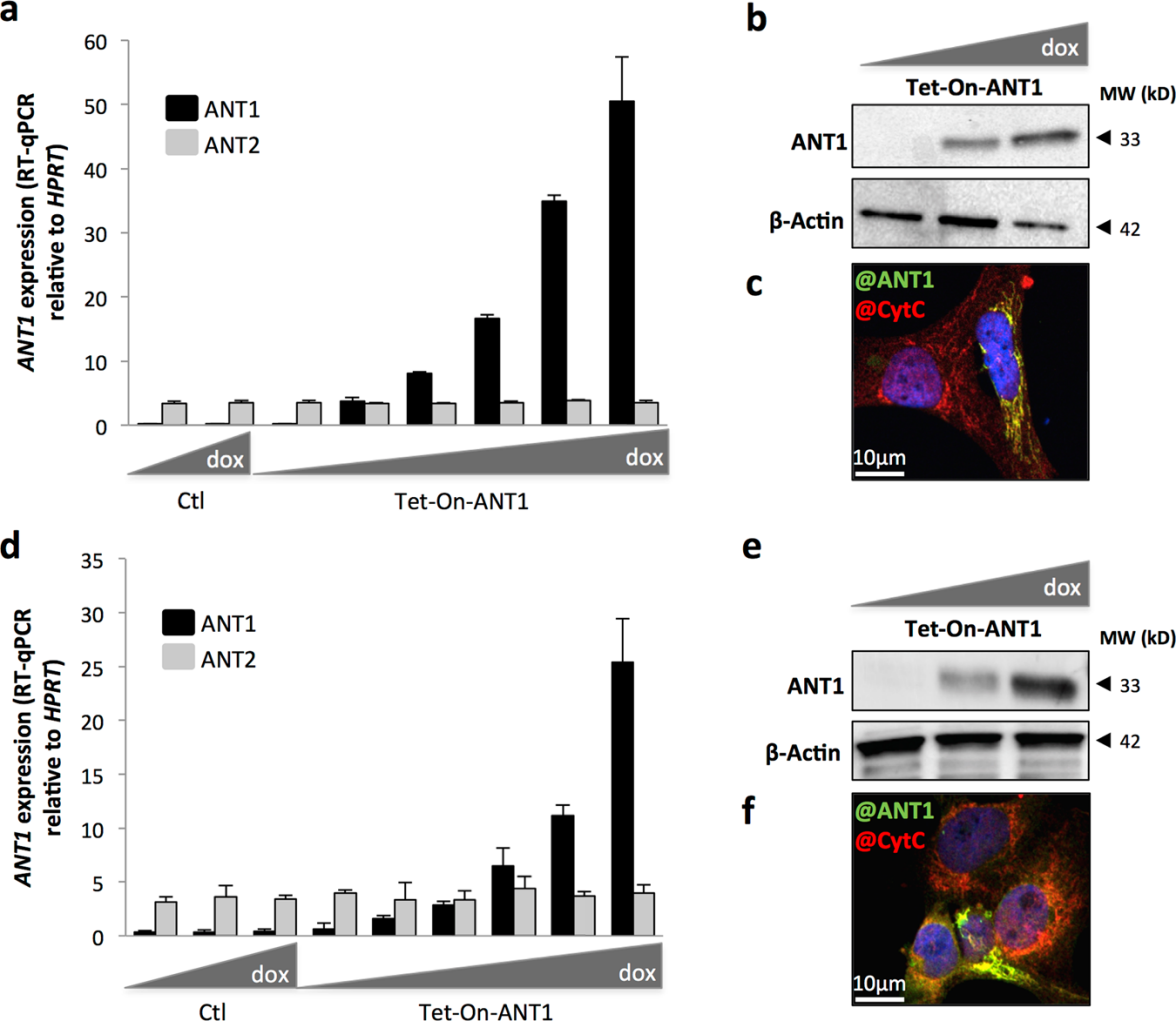
Efficiency of the inducible Tet-On strategy used to induce ANT1 expression in RMS cells. Efficiency of *ANT1* induction by Tet-On system in RH30 (**a**–**c**) and RD (**d**–**f**) cells, 48 h after doxycycline treatment, as shown by quantification of *ANT1* expression by RT-qPCR, relatively to the housekeeping gene *HPRT* (**a**, **d**), immunoblot with actin as a loading control (**b**, **e**), and immunofluorescence showing colocalization with the cytochrome c mitochondrial marker (**c**, **f**).

**Fig. 5:**
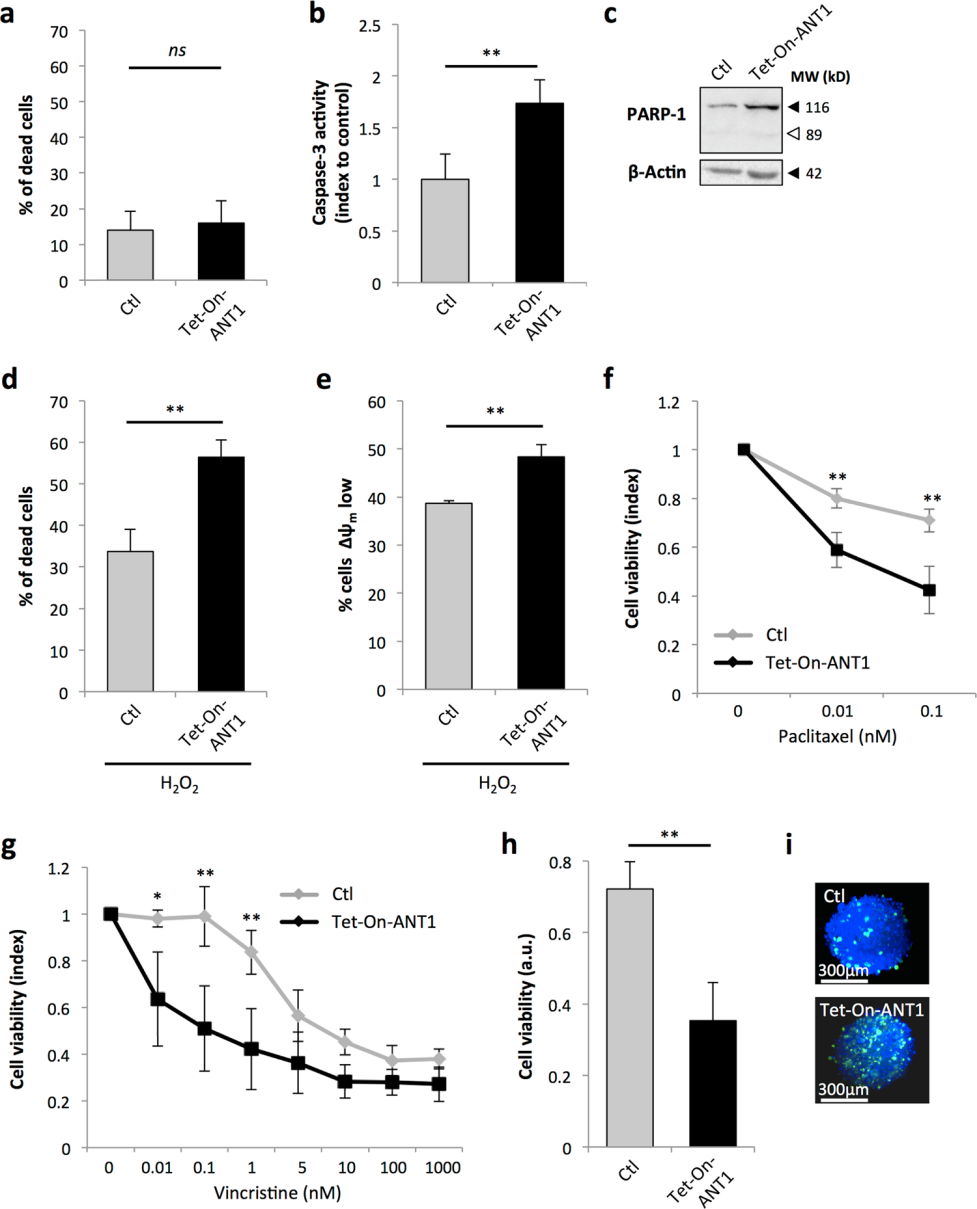
Restoration of ANT1 is sufficient to sensitize RMS cells to death induced by stress and chemotherapy. **a** Restoring ANT1 expression using an inducible Tet-On system (RH30^High^ cells) does not change the percentage of dead cells. Dead cells were stained by DAPI and quantified by image cytometry. Results are presented as means ± s.d.; *n* = 3. ns not significant; two-sided independent samples *T*-test. **b** Increase in basal caspase-3 activity in RH30^High^ cells. Results are presented as means ± s.d.; *n* = 3. **p < 0.01; two-sided independent samples *T*-test. **c** Absence of PARP-1 cleavage in RH30^High^ cells in normal growth conditions. Actin is used as a loading control. Increase in the percentage of dead cells (**d**) and with loss of ∆ψ_m_ (**e**) in RH30^High^ cells upon H_2_O_2_ treatment, compared to control cells. Quantification was made by image cytometry using DAPI and JC-1 staining, respectively. Results are presented as means ± s.d.; *n* = 3. **p < 0.01; two-sided independent samples *T*-test. Increased sensitivity of RH30^High^ cells to 24 h of Paclitaxel (**f**) or Vincristine (**g**) treatment, compared to control cells (WST-1 assay). Results are presented as means ± s.d.; *n* = 3. *p < 0.05, **p < 0.01; two-sided independent samples *T*-test. **h** Increased sensitivity of RH30^High^ 3D spheroids to Paclitaxel treatment, compared to control 3D structures (CellTiter-Glo^®^ assay). Results are presented as means ± s.d.; *n* = 5. **p < 0.01; two-sided independent samples *T*-test. **i** Caspase-3 activity measured by immunofluorescence. Spheroids were imaged by SPIM after active caspase-3 (green) and DAPI (blue) immunostaining. Representative images are shown.

As the loss of ANT1 conferred selective advantages to RMS tumor cells, we wondered whether restoration of its expression may have tumor suppressive effects. To this end, we designed Tet-On inducible stable RD and RH30 cell lines to assess the impact of the restoration of *ANT1* expression on stress-induced tumor cell death (Fig. 4a–f). The RH30 line was chosen for this rescue experiment, as these cells showed the lowest levels of *ANT1* expression. The two induced cell lines will be further designated as RD^High^ and RH30^High^. As ANT1 overexpression drives apoptotic cell death^40^, we chose to use a doxycycline dose that was sufficient to restore *ANT1* expression without affecting tumor cell death in normal growth conditions (Fig. 5a). In this steady-state condition, we observed an increase in caspase-3 activity in RH30^High^ cells, independently of any death stimuli (Fig. 5b). In accordance with the observation that cell viability was not affected by restoring *ANT1* expression, we did not detect, in these basal conditions, changes in PARP cleavage, as a downstream effector of the apoptotic pathway (Fig. 5c). We then wondered whether these cells may be more sensitive to stress. Restoring *ANT1* expression was indeed sufficient to increase cell sensitivity to H_2_O_2_ treatment in both RH30^High^ and RD^High^ lines (Fig. 5d and Fig. S2a). Increase in cell death in RH30^High^ cells upon H_2_O_2_ treatment was accompanied by an increase in the percentage of cells with mitochondrial membrane depolarization (Fig. 5e). These results reveal that *ANT1* re-expression is sufficient to restore cell sensitivity to stress-induced death in RMS cell lines.

We then examined whether ANT1 restoration may also counteract the resistance of RMS cells to chemotherapy. Following treatment with Paclitaxel, we observed that viability of RH30^High^ cells was reduced compared to control cells (Fig. 5f and Fig. S2b). Similar results were obtained with Vincristine (Fig. 5g). To confirm these results in a more relevant system, we set up 3D-spheroid RMS models using both RH30 control and RH30^High^ RMS cells. We observed that re-expression of *ANT1* in this 3D-system was also sufficient to decrease resistance to chemotherapy (Fig. 5h). This effect likely involved induction of apoptosis via MPT since it was associated with an increase in caspase-3 activity (Fig. 5i). Altogether, these results indicate that ANT1 expression may prime cells to death by modulating caspase-3 activity and may thereby affect their ability to resist stress. Inversely, loss of ANT1 may trigger an increased resistance to stress and to chemotherapies conventionally used for RMS.

## Discussion

Mitochondria are a global hub for intracellular signaling, regulating energy production, metabolism, stress, or apoptotic response to stimuli for example. Previous reports pointed to a pivotal role of ANT1 in some of these processes, especially for the control of OXPHOS metabolism and the induction of MPT-regulated cell death. Here, we unveil that loss of *ANT1* expression is sufficient to perturb mitochondria functioning at different levels, thereby supporting oncogenic properties of RMS tumor cells.

Myogenesis involves successive reprogramming of myogenic progenitors, which switch from a dormant to a proliferative state, before differentiating into mature myofibers or entering quiescence (self-renewal). Differentiation requires the acquisition of a metabolism able to sustain the high energetic cost of muscle contraction^36,43^ and is accompanied with an increased resistance to apoptosis. *ANT1* expression is suggested to increase upon differentiation, to sustain the switch from a glycolytic metabolism observed in myoblasts to an oxidative one in myofibers^11^. We observed here that *ANT1* expression is low in RMS tumors, and is notably reduced compared to fetal muscles, which are frequently used as their non-tumoral equivalents^44,45^. Since the precise origin of RMS cells is not clearly defined and may result from the oncogenic transformation of different myogenic precursors, it is unclear whether the low levels of *ANT1* expression correspond to the maintenance of the signature observed in early myogenic precursors or to a secondary loss arising during tumor transformation. However, our results support the view that increased expression of *ANT1* during myogenesis is not only a consequence of muscle cell differentiation but that it may also contribute to this process. In particular, ANT1 may influence muscle cell commitment, by modulating caspase-3 steady-state activity, which is a determinant factor during myogenesis^39,46–48^. Perturbing specific metabolic pathways has also been shown to affect myogenic cell fate. We provided here evidence that reduction in *ANT1* expression promotes metabolic switch, which may also limit the commitment of RMS cells. Hence, low levels of *ANT1* may drive a change of state in RMS cells displaying immature proliferative properties, corresponding somehow to early steps of muscle regeneration, by acting via different processes.

In this study, we established that low expression levels of *ANT1* confer selective advantages to RMS tumor cells. Few studies have been dedicated to characterizing the precise role of ANT1 in cancers, due to the high level of homology between ANT1 and ANT2, which precludes their distinction. Moreover, apparently contradictory results have been reported in the two studies published so far on the role of ANT1 in tumorigenesis. Indeed, in these two independent publications, both *ANT1* silencing^42^ and its overexpression^40^ led to reduced survival of glioblastoma cells and breast cancer cells, respectively. Our results reconcile these observations by showing that ANT1 acts as a tumor suppressor gene, likely by supporting cell commitment to a differentiation process, via changes in metabolic and death-associated signaling, but especially by priming cells to death and increasing their sensitivity to stressful conditions. However, total loss of ANT1 may have deleterious effects notably by preventing metabolic plasticity of tumor cells and by increasing the oxidative stress level beyond a toxic threshold. Hence, optimal low levels of ANT1 likely exist that strengthen the oncogenic properties of tumor cells. Perturbing this tight equilibrium appears to be an appealing targeted therapeutic strategy in RMS treatment.

## Material and methods

### Patients and RNA samples

RMS (*n* = 67) and adult (*n* = 5), fetal (*n* = 9), DMD (*n* = 5), BD (*n* = 5), and denervated (*n* = 5) muscle biopsies were collected by Centre Léon Bérard and Hospices Civils de Lyon (France) Biological Resources Centres, respectively. Tissue banking and research were conducted according to national ethics guidelines, after obtaining the written informed consent of patients. For mRNA extraction, 10-µm-thick FFPE sections were deparaffinized, lysed in 200 µL of ATL buffer (Qiagen) with 2 µL Proteinase K (Roche). In total, 1 mL of Trizol (Life Technologies) was then added to isolate total RNA, before classical purification using 200 µL chloroform and 500 µL isopropanol with 1 µL of Glycoblue (ThermoFisher). After washing in ethanol 75%, digestion of contaminant genomic DNA was performed for 1 h at 37 °C by resuspending the nucleic acid pellets in 15 µL of 1× buffer containing 0.25 µL of 100 mM dithiothreitol (DTT), 2 µL of 1 M DNase, and 1 µL of RNAsin. A new step of precipitation by isopropanol/ethanol was then performed to eliminate DNase and RNA samples were frozen (−80 °C) until further use.

### 2D and 3D cell cultures and design of cell models

In vitro studies were conducted using the human RD ERMS cells (ATCC), and the RH30 ARMS cells (DSMZ). RD and RH30 cells were routinely maintained under standard conditions (37 °C and 5% CO_2_ in humidified incubator) in Dulbecco’s Modified Eagle’s Medium (DMEM) supplemented with 10% fetal bovine serum (FBS) or in Roswell Park Memorial Institute medium supplemented with 10% FBS, respectively. 3D spheroids were obtained by seeding 2.5 × 10^3^ RH30 cells onto 96-well low-attachment plates with 0.5% Matrigel for 3 days.

For stress induction, cells were exposed to 0–1200 μM (RD), 0–75 µM (RH30) of H_2_O_2_ for 6 h or to Paclitaxel or Vincristine as chemotherapies for 48 h (see concentrations range in Figures).

To generate stable knockdown cell lines, a conditional CRISPR-Cas9-KRAB system was used. In this system, Cas9 and KRAB from *Streptococcus pyogenes* are placed under the control of a tetracycline-inducible promoter in pHAGE TRE dCas9-KRAB plasmid, conferring a G418 resistance to cells. Human *SLC25A4* (further referred to as *ANT1*) coding sequence (NM_001151.4, ENST00000281456.11) was cloned at BfuA1 sites in the pLKo.1-puro U6 sgRNA BfuA1 stuffer, conferring puromycin resistance to cells. These plasmids were a gift from Rene Maehr and Scot Wolfe (Addgene plasmids #50917 and #50920)^49^. RD and RH30 cell lines were then transduced with both plasmids thanks to lentivirus and using lipofectamine, according to the manufacturer’s instructions. Clones were then selected for stable integration for 10 days in G418 (RD: 1 mg/mL; RH30: 600 µg/mL) and puromycin-containing medium (RD: 0.6 µg/mL; RH30: 0.4 µg/mL). All stable knockdown cells were further tested to check *ANT1* expression in response to doxycycline. Control cells were transduced with the pHAGE TRE dCas9-KRAB plasmid alone to exclude any Cas9 off-target effects. All experiments presented in this paper were performed 48 h after doxycycline induction.

To generate inducible stable cell lines, the human *ANT1* coding sequence was cloned at Sal1 and Cla1 sites into the pITR plasmid (gift from Prof. Rolf Marschalek, Goethe-University of Frankfurt, Germany), a sleeping beauty-based vector allowing the doxycycline-inducible expression of *ANT1*, using zeocin resistance gene and red fluorescent protein (mCherry) as selection markers. RD and RH30 cell lines were transfected with *ANT1*-pITR or an empty plasmid as a control, and a sleeping beauty transposase expression vector (SB100X). Clones were then selected for stable integration for 10 days in zeocin-containing medium. Control and ANT1-positive clones that stably integrated those constructs were selected by flow cytometry using mCherry as a marker. All stable cells were further tested to check *ANT1* expression in response to doxycycline. These cell lines had recently been tested negative for mycoplasma. All experiments presented in this paper were then performed 24 h after doxycycline induction.

### Quantitative RT-PCR

Total RNA from cell lines was extracted using the Nucleospin RNAII kit (Macherey-Nagel) and 1 µg was reverse-transcribed using the iScript cDNA Synthesis kit (BioRad), according to the manufacturer’s instructions. Expression of *ANT1* [forward primer (5′ CAAGGGGATGCTGCCTGACC 3′) and reverse primer (5′ GGACTGCATCATCATTCTACG 3′)], *ANT2* [forward primer (5′ CACTGCAAAGGGAATGCTTCCGG 3′) and reverse primer (5′ GTACATGATGTCAGTTCCTTTGCG 3′)], *MYOG* [forward primer (5′ TGCCATCCAGTACATCGAGC 3′) and reverse primer (5′ GCAGATGATCCCCTGGGTTG 3′)], *TNNT3* [forward primer (5′ CCTGGCCAAGGCTGACCAG 3′) and reverse primer (5′ CCTTGGCCTTGTCCCTCAG 3′)], and *MYBPC1* [forward primer (5′ GGCACAGTCGGGTGTACAC 3′) and reverse primer (5′ CCCAGTAGATTCGTGCACTTC 3′)] were assessed by real-time quantitative RT-qPCR on a LightCycler^®^ 480 instrument (Roche) using the LightCycler^®^ 480 SYBR Green I Master Mix, according to the manufacturer’s instructions (Roche). The ubiquitously expressed *HPRT1* [forward primer (5′ TGACACTGGCAAAACAATGCA 3′) and reverse primer (5′ GGTCCTTTTCACCAGCAAGCT 3′)] was used as an internal calibrator.

### Cell viability, proliferation, and death assays

For image cytometry, cells were seeded onto six-well plates at a density of 1 × 10^5^ cells/well and treated with doxycycline to modulate *ANT1* expression. Cell number and viability were quantified by image cytometry on a NucleoCounter NC-3000 (Chemometec, Allerød, Denmark) according to the procedure provided by the manufacturer, using a co-staining of Acridine Orange (to quantify total number of cells) and DAPI.

For WST-1, cells were seeded onto 96-well plates at 8000 cells/well. After 24, 48, and 72 h doxycycline induction, assays were performed using Cell Proliferation Reagent WST-1 (Cat No.11644807001; Roche), according to the manufacturer’s instructions. Thereafter, the number of viable metabolically cells was quantified based on the absorbance of formazan formed at 450 nm, analyzed using a microplate reader (Tecan Infinite^®^ M1000 PRO).

For 3D spheroids, cell viability was measured by using the CellTiter-Glo^®^ Luminescent Cell Viability Assay (Promega, Madison, Wisconsin), according to the manufacturer’s instructions. This assay is based on the quantification of the ATP produced, reflecting the presence of metabolically active cells. Luminescence was then measured using a microplate reader (Tecan Infinite^®^ M1000 PRO).

Mitochondrial potential measurements were carried out thanks to the fluorescent-based Mitochondrial Potential Assay (Chemometec) according to the following procedure. Cells were seeded onto six-well plates at a density of 1 × 10^5^ cells/well. After 24 h, they were treated with doxycycline, and eventually with H_2_O_2_ or chemotherapy after induction of CRISPR-Cas9/Tet-On systems. Cells were then collected and washed with 1 mL PBS 1×. Each sample was resuspended in 12.5 μL of JC-1 solution (Solution 7, Chemometec). The JC-1 lipophilic cationic dye has dual-fluorescent properties, differentiating healthy and cells in which MPT-regulated cell death is induced. In healthy cells, the intact mitochondrial membrane potential facilitates the accumulation of JC-1 in the mitochondria, becoming red fluorescent. On the contrary, in cells in which MPT-regulated cell death is engaged, the mitochondrial potential collapses and JC-1 localizes to the cytosol in its monomeric green fluorescent form. After incubation at 37 °C for 10 min, stained cells were centrifuged at 400 × *g* for 5 min at room temperature (RT) and the supernatant was then completely removed without disturbing the cell pellet. Cell pellet was rinsed twice in 1 mL PBS 1× and then resuspended in 0.25 mL of DAPI solution (Solution 8, Chemometec). Each population of cells stained with these different dyes was then quantified by image cytometry using the NucleoCounter NC-3000 (Chemometec).

Caspase-3 activity assay was performed using the Caspase-3 Fluorimetric Assay Kit (Biovision K105-400), according to the manufacturer’s instructions. Briefly, 2 × 10^5^ cells were seeded onto six-well plates and treated with doxycycline for 24 h (inducible stable cell lines) or 48 h (stable knockdown cell lines). Cells were then collected and resuspended in 55 µL of cell lysis buffer and incubated on ice for 30 min. After centrifugation, 50 µL of sample were loaded into a 96-well plate and mixed with 50 µL of reaction buffer. Fluorescence generated by caspase related activity was quantified by fluorescent detection of free AFC after cleavage from the peptide substrate DEVD-AFC using a microplate reader (Tecan Infinite^®^ M1000 PRO). A Bradford protein assay was performed to normalize caspase activity against the total protein amount of the sample.

For 3D cultures, active caspase-3 was defined by immunostaining using Cleaved Caspase-3 (9661S, cell signaling) specific antibody. Briefly, spheroids were collected, fixed, permeabilized (4% paraformaldehyde, PBS 1×/Triton 0.1%) and blocked in PBS BSA 3% for 45 min. Primary antibody was added (diluted 1/200 in PBS 1×) overnight at 4 °C. Secondary antibody was added (diluted 1/500 in PBS 1×) for 1 h with DAPI at RT.

### Western blot and co-immunofluorescence

Cells were lysed in 200 µL of 2× Laemmli Sample Buffer (BioRad, Hercules, California) containing 100 mM DTT. Protein extracts were then analyzed by immunoblot. Briefly, proteins were loaded into 10% SDS‐polyacrylamide gels and blotted onto PVDF sheets (BioRad) using the TurboBlot technology (BioRad). Membranes were blocked with 5% non‐fat dried milk or 5% BSA in TBS/0.1% Tween 20 (TBS‐T) for 1 h and then incubated overnight with anti-ANT1/2 (sc-9299, Santa Cruz), anti-P-ERK1/2 (4370, Cell signaling), anti-ERK1/2 (9102, Cell signaling), anti-P-mTOR (2971, Cell signaling), anti-mTOR (2972, Cell signaling), anti-P-S6 (2211, Cell signaling), anti-S6 (2217, Cell signaling), anti-PARP-1 (9542, Cell signaling), anti-α-tubulin (ab15246, Abcam), or anti-β-actin (mAB1501R, Sigma). After three washes with TBS-Tween 0.1%, filters were incubated with the appropriate HRP-conjugated secondary antibody (1:10 000, Jackson ImmunoResearch) for 1 h. Detection was performed using the ECL Chemiluminescence System (Pierce). Membranes were imaged on the ChemiDoc Touch Imaging System (BioRad).

Expression of ANT1 and cytochrome c was assessed by co-immunofluorescence on cells fixed 20 min in 4% paraformaldehyde and permeabilized in PBS 1×/Triton 0.2%, using specific antibodies (respectively sc-9299, Santa Cruz, 1:500; and ab90529, Abcam, 1:500). Fluorescence labeling was obtained using corresponding secondary antibodies coupled to Alexa Fluor-488 or Alexa Fluor-647 at a dilution of 1:500 (Invitrogen).

### Metabolic analyses

NMR-based metabolomic analyses were carried out on intact cells using the HRMAS (High Resolution Magic Angle Spinning-Nuclear Magnetic Resonance) method (IRMaGe facility, CEA-Grenoble). After washing 1 × 10^6^ cells with D2O (Deuterium Oxide, Cortecnet), cell pellet was inserted into disposable inserts (Cortecnet), as described previously^50^. Inserts were sealed and frozen into liquid nitrogen and stored at −80 °C until further analyses. All ^1^H HRMAS MR spectra were acquired on a Bruker Avance III spectrometer (IRMaGe, CEA, Grenoble, France) at 500 MHz^50^. Samples were spun at 4000 Hz and temperature maintained at 4 °C for all experiments. One-dimensional spectra were acquired using a Carr–Purcell–Meiboom–Gill pulse sequence (TE = 30 ms, 256 averages, 17 min). The residual water signal was pre-saturated during 1.7 s of relaxation. Obtained spectra were all processed for multivariate statistics using NMRProcFlow 1.2 online (https://nmrprocflow.org/).

Simultaneous multiparametric metabolic analyses of live cells was performed in the Seahorse XF24^®^ extracellular flux analyzer (Seahorse Bioscience, USA). RH30 and RD cells were seeded in XF24 V7 multi-well plates (1.5 × 10^4^ cells per well) for 5 h at 37 °C in 5% CO_2_. One hour before recording the glycolytic activity, cell culture medium was replaced with minimal DMEM (0 mM glucose) without phenol red supplementation with 143 mM NaCl, 2 mM glutamine, and 1 mM sodium pyruvate, pH 7.4. ECAR was measured under these basal conditions and after sequential injections of glucose (10 mM), the ATP synthase inhibitor oligomycin (1 μM), and the glycolysis inhibitor 2-deoxyglucose (100 mM). To record the mitochondrial activity, the same assay medium was used and supplemented with 1 mM sodium pyruvate and 10 mM glucose. OCR was analyzed before and after sequential injections of oligomycin (1 μM), the electron transport chain uncoupler FCCP (1 μM), and specific inhibitors of the mitochondrial respiratory chain antimycin A/rotenone (0.5 μM). To normalize OCR and ECAR data against cell number, cells were fixed with glutaraldehyde 1%, stained with crystal violet 0.1% in methanol 20% (Sigma-Aldrich), which was finally solubilized in DMSO to measure dye absorption at 600 nm on a microplate reader (Tecan Infinite® M1000 PRO). SRC was calculated as the difference between maximal respiration and basal respiration as follows: [(OCR after FCCP injection) − (OCR before oligomycin injection)] × 100.

### Detection of ROS using CellROX^®^ staining

CellROX^®^ green oxidative stress reagent (Invitrogen, C10444), which is non-fluorescent while in a reduced state and exhibits a strong fluorogenic signal upon oxidation, was used to detect ROS. A total of 5 × 10^3^ cells were seeded onto 96-well plates and treated with 1000 μM H_2_O_2_ after 48 h doxycycline induction. CellROX^®^ green was then added in each well at a final concentration of 500 ng/mL and incubated for 15 min. Fluorescence intensity was then monitored with an Incucyte ZOOM^®^ system (Essen BioScience, Michigan, USA). Phase images were obtained every 2 h for 24 h.

### Statistical analysis

Prism 7.0 (GraphPad) was used for statistics. All data are representative of at least three independent repeats if not otherwise stated. The letter *n* refers to the number of independently performed experiments representative of the data shown in the figures. The statistical significance in this study was determined by two-sided independent samples *T*-test, with Welch’s correction in cases the variances of the populations are not equal. A *p* value *p* < 0.05 was considered to be significant. Statistical significance was described as follows: *p < 0.05, **p < 0.01, ***p < 0.001, ****p < 0.0001. The error bars represent the standard deviation (s.d.).

## Supplementary information

Supplementary information

Supplementary Figure 1

Supplementary Figure 2
