# Supplementary material for: Low expression of ANT1 confers oncogenic properties to rhabdomyosarcoma tumor cells by modulating metabolism and death pathways
Source: Cell Death Discov. 2020 Jul 24;6:64. doi: 10.1038/s41420-020-00302-1 (PMC7382490; doi:10.1038/s41420-020-00302-1)
Supplement: Supplementary file 1 — Supplementary information [file 41420_2020_302_MOESM1_ESM.doc]

**SUPPLEMENTARY INFORMATIONS**

**Supplementary Figure 1: Characterization of ANT1-associated properties in RMS**

**a.** Prognostic value of *ANT1* expression in fusion-negative RMS (n = 58), corresponding to ERMS and ARMS that do not present Pax3/7-FOXO1 translocation. Cut-off between *ANT1* high and low group corresponds to the 1^st^ quartile of expression. Data were obtained from the E-TABM-1202 dataset. **b-c.** *ANT1* expression in RMS biopsies (n = 147) is negatively correlated with two markers of proliferation (b) KI67, and (c) CDC7. R2 cancer analysis, Davicioni E-TABM-1202 dataset. **d.** Specificity of *ANT1* silencing by CRISPR-Cas9 in RD cells, 48 h after doxycycline treatment. There was no change in *ANT2* (*SLC25A5*) expression quantified by RT-qPCR, relatively to the housekeeping gene *HPRT*. Results are presented as means +/- s.d.; n = 3. **e.** Increase in viable cell concentration in *ANT1* CRISPR-Cas9 RD cells (RD^Low^), 48 h after silencing of *ANT1* expression by doxycycline treatment (image cytometry quantification, DAPI/Acridine orange co-staining). Results are presented as means +/- s.d.; n = 3. *: p < 0.05; two-sided independent samples T-test. **f.** Efficiency of siRNA targeting *ANT1* in myoblasts. Results are presented as means +/- s.d. of 3 RT-qPCR, relatively to the housekeeping gene *HPRT*; n = 3. **g.** Increase in viable myoblasts concentration, 48 h after silencing of *ANT1* using a siRNA strategy. Results are presented as means +/- s.d.; n = 3. **: p < 0.01; two-sided independent samples T-test. **h.** Decreased sensitivity of RD^Low^ cells to Vincristine after 72 h of treatment, compared to control cells (WST-1 assay). Results are presented as means +/- sem ; n = 3. *: p < 0.05; two-sided independent samples T-test.

**Supplementary Figure 2: Impact of ANT1 expression on RMS cell death**

**a.** Increased sensitivity to death of RH30^High^ cells treated for 6 h with H_2_O_2_. Results are presented as means +/- s.d.; n = 3. DAPI staining quantified by image cytometry (NucleoCounter). **: p < 0.01; two-sided independent samples T-test. **b.** Decreased viability of RD^High^ cells treated for 24 h with Paclitaxel, 24 h after induction of *ANT1* expression by doxycycline treatment, compared to control cells (WST-1 assay). Results are presented as means +/- s.d.; n = 3. *: p < 0.05; two-sided independent samples T-test.
