# Supplementary figures and images for: Low expression of ANT1 confers oncogenic properties to rhabdomyosarcoma tumor cells by modulating metabolism and death pathways
Source: Cell Death Discov. 2020 Jul 24;6:64. doi: 10.1038/s41420-020-00302-1 (PMC7382490; doi:10.1038/s41420-020-00302-1)

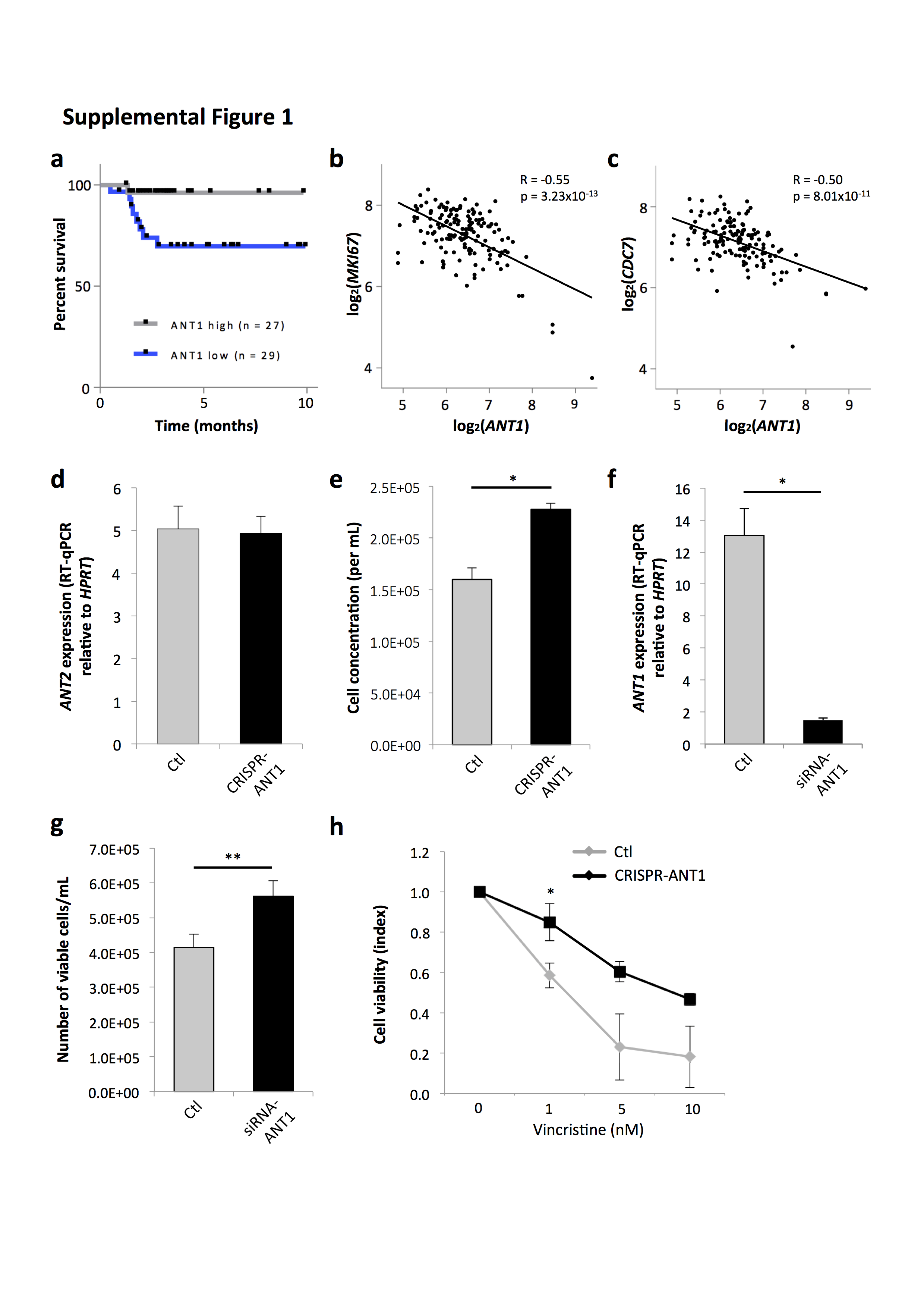

Supplement: Supplementary file 2 — Supplementary Figure 1 [file 41420_2020_302_MOESM2_ESM.png]

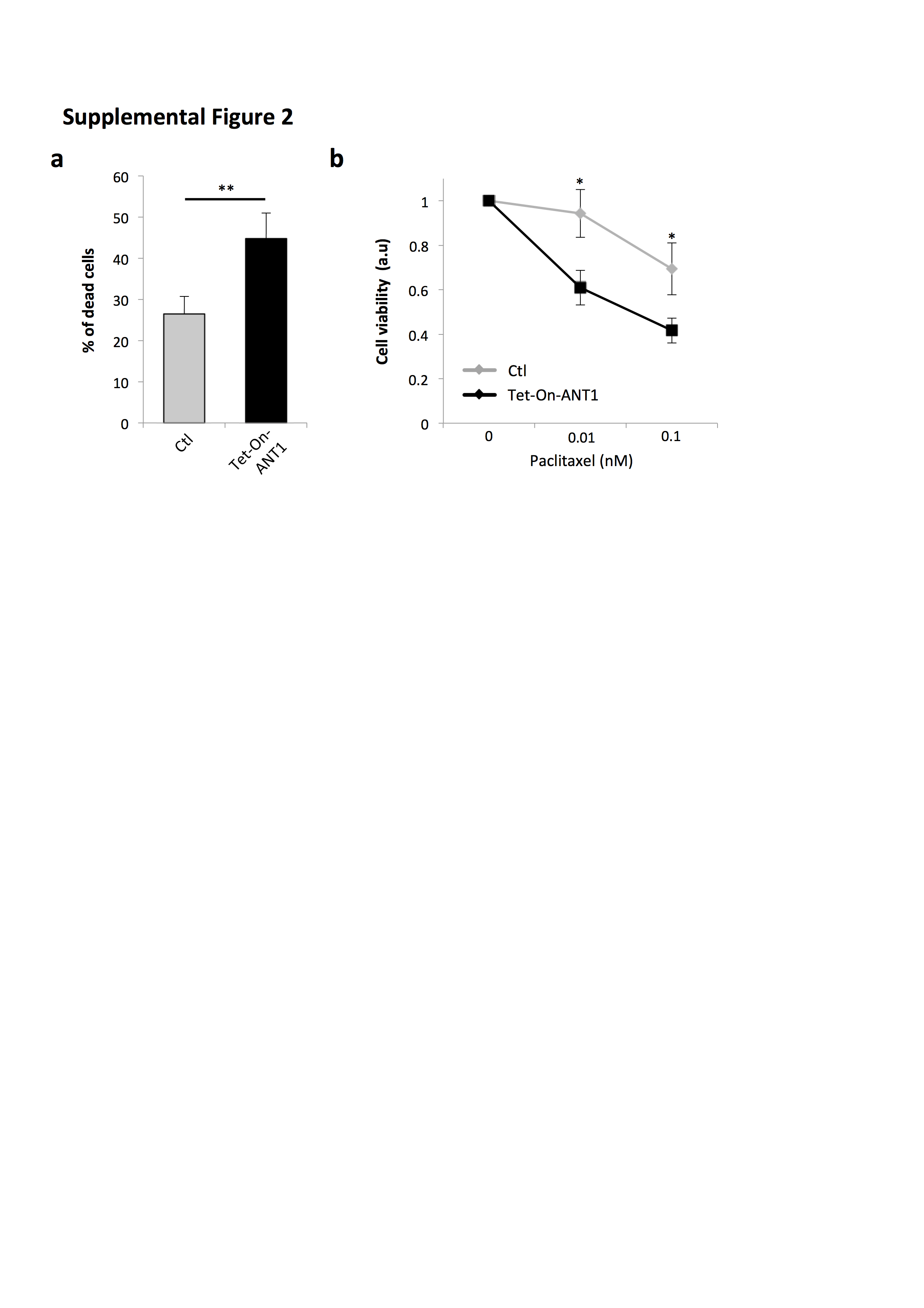

Supplement: Supplementary file 3 — Supplementary Figure 2 [file 41420_2020_302_MOESM3_ESM.png]
